# Modification of the moving point test method for nighttime eddy CO_2_ flux filtering on hilly and complex terrains

**DOI:** 10.1016/j.mex.2019.05.012

**Published:** 2019-05-14

**Authors:** Minseok Kang, Joon Kim, Bindu Malla Thakuri, Junghwa Chun, Chunho Cho

**Affiliations:** a National Center for AgroMeteorology, Seoul, South Korea; b Program in Rural Systems Engineering, Department of Landscape Architecture & Rural Systems Engineering, Seoul National University, Seoul, South Korea; c Interdisciplinary Program in Agricultural & Forest Meteorology, Seoul National University, Seoul, South Korea; d Institute of Green Bio Science and Technology, Seoul National University Pyeongchang Campus, Pyeongchang, South Korea; e Research Institute for Agriculture and Life Sciences, Seoul National University, Seoul, South Korea; f Department of Environmental Planning, Graduate School of Environmental Studies, Seoul National University, Seoul, South Korea; g Department of Forest Conservation, National Institute of Forest Science, Seoul, South Korea; h National Institute of Meteorological Sciences, Seogwipo, South Korea

**Keywords:** Modified moving point test method, Eddy covariance, Advection, Nighttime CO_2_ flux correction, Moving point test, Hilly and complex terrain

## Abstract

The measurement of carbon dioxide (CO_2_) fluxes using the eddy covariance technique is difficult in forests in complex terrain because of the horizontal advection of CO_2_ at night. This results the under- or overestimation of net ecosystem exchanges of CO_2_. We propose a technique for nighttime filtering (and correction) of CO_2_ fluxes to eliminate (and replace) those significantly affected by horizontal advection: the modified moving point test method. This was developed by merging the friction velocity filtering and van Gorsel methods. It is based on an approach using moving windows for time and friction velocity, allowing a nighttime CO_2_ flux correction that includes an assessment of CO_2_ drainage at midnight. We tested the method using datasets from two flux towers in forests in hilly and complex terrains, where the application of generic nighttime filtering methods is difficult because CO_2_ drainage is generated earlier than the time assumed by the generic methods. The method produced carbon budgets consistent with previous research results, while showing improved applicability.

• We propose a nighttime CO_2_ flux filtering method for hilly and complex terrain that combines the friction velocity filtering and van Gorsel methods.
• This method determines the friction velocity threshold and the significance of CO_2_ drainage at midnight based on an approach using moving windows for time and friction velocity.
• The method produced consistent results and shows improved applicability.

## Graphical abstract

**Figure:**
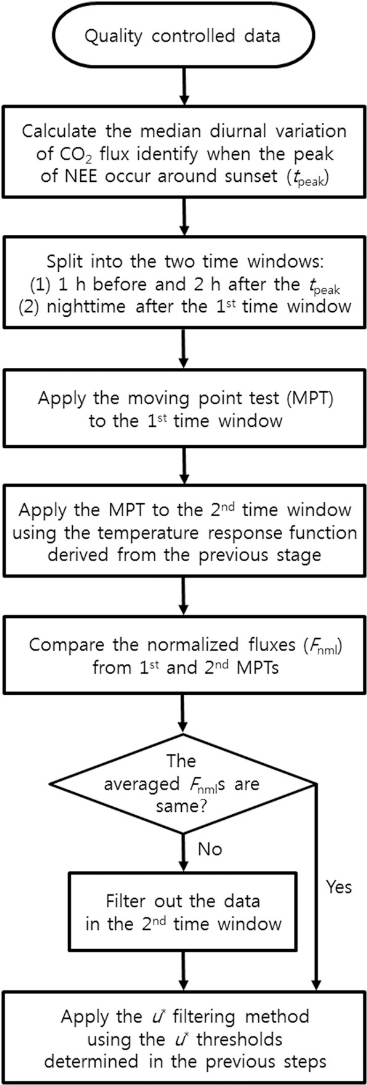

**Specifications Table**

**Table:** 

Subject Area:	*Earth and Planetary Sciences*
More specific subject area:	*Biogeosciences*
Method name:	*Modified moving point test method*
Name and reference of original method:	*Original moving point test method [Gu, L.H., Falge, E.M., Boden, T., Baldocchi, D.D., Black, T.A., Saleska, S.R., Suni, T., Verma, S.B., Vesala, T., Wofsy, S.C., Xu, L.K., 2005. Objective threshold determination for nighttime eddy flux filtering. Agric. Forest Meteorol. 128, 179–197]* Original van Gorsel method [van Gorsel, E., Leuning, R., Cleugh, H.A., Keith, H., Suni, T. 2007. Nocturnal carbon efflux: reconciliation of eddy covariance and chamber measurements using an alternative to the u*-threshold filtering technique. Tellus B 59, 397–403]
Resource availability:	*MATLAB script for applying the method and sample data*

## Method details

### Background – eddy covariance technique

The eddy covariance (EC) technique is a micrometeorological measurement method to monitor the vertical turbulent transport of mass and energy between the surface and atmosphere using both a fast response (> 10 Hz) sonic anemometer-thermometer and gas analyzer placed on an observation tower. The net ecosystem exchange (NEE) of carbon dioxide (CO_2_) can be expressed as [e.g., [1,2]]:

(1) $$\text{NEE}=\underset{\text{I}}{\underset{︸}{{\left(\bar{w'c'}\right)}_{\begin{array}{l} h \end{array}}}}+\underset{\text{II}}{\underset{︸}{\int_{0}^{h}\left[\frac{\partial \bar{c}}{\partial t}\right]dz}}+\underset{\text{III}}{\underset{︸}{\int_{0}^{h}\bar{w(z)}\left[\frac{\partial \bar{c}}{\partial z}\right]dz}}+\underset{\text{IV}}{\underset{︸}{\int_{0}^{h}\left(\bar{u(z)}\frac{\partial \bar{c}}{\partial x}+\bar{v(z)}\frac{\partial \bar{c}}{\partial y}\right)dz}}$$

where *c* is the CO_2_ concentration; *u, v*, and *w* represent the velocity components in the longitudinal (*x*), lateral (*y*), and vertical (*z*) direction, respectively; *h* is the measurement height; an overbar denotes Reynolds averaging; a prime is the deviation from the mean; and *t* is time. The term I (i.e., eddy flux) represents the flux via vertical turbulence, the term II (i.e., storage flux) is the flux stored below the measurement height, the term III (i.e., vertical advective flux) is the flux advected by the mean vertical flow in the presence of a vertical CO_2_ gradient, and the term IV (i.e., horizontal advective flux) represents the fluxes transported by the horizontal mean flow and turbulence in the presence of a horizontal CO_2_ gradient beneath the height of measurement. Assuming that the site is flat and homogeneous, and under the well-developed turbulent condition (III ≈ IV ≈ 0), NEE can be quantified as the sum of the terms I and II.

### Motivation

For EC measurements taken over complex mountainous terrain, nighttime CO_2_ flux correction is one of the most critical and challenging tasks. The nighttime CO_2_ flux correction filters out the advection-affected data (i.e., NEE ≠ I + II) from measurements taken at night and fills any gaps. There are two widely used methods: (1) the friction velocity (u*) filtering method, and (2) the filtering method based on the peak CO_2_ flux near sunset. u* is measured simultaneously with CO_2_ flux, which is defined as:

(2) $$u^{*}={\left({\bar{u'w'}}^{2}+{\bar{v'w'}}^{2}\right)}^{1/4}$$

**Fig. 1:**
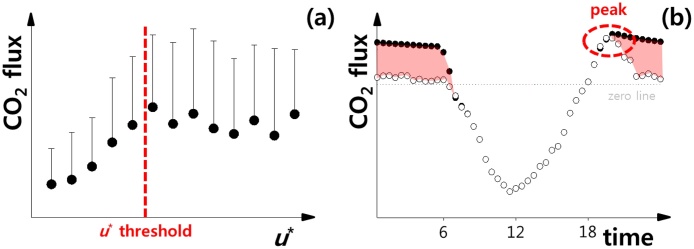
Simplified (a) dependency of measured ecosystem respiration (i.e., nighttime measured CO_2_ flux) on friction velocity (u*) with u* threshold (red dashed line) and (b) diurnal variation in the accurate net ecosystem exchange (black dot) and measured CO_2_ flux (white dot) if the flux is underestimated due to a non-turbulent evacuation of CO_2_ for a typical site [adapted from Kang et al. [9], Aubinet et al. [11]]. The red surface indicates the difference between the accurate and the measured.

The most commonly used method is the u* filtering method that optimizes the parameters of the ecosystem respiration (ER) function using observed nighttime CO_2_ fluxes when u* is greater than a threshold (i.e., there is no dependency of the nighttime CO_2_ flux on u*, Fig. 1 a) [e.g., [3,4]]. The filtered data are replaced with the estimated data using ecosystem temperatures and the ER function with the optimized parameters. The u* filtering method cannot be applied at sites where the u* threshold cannot be determined, and/or drainage flow is generated during most of the night. Accordingly, an alternative method using time as a parameter for filtering was developed for hilly terrain sites that are affected by drainage flow (i.e., where the u* filtering method with a typical u* threshold such as 0.25 m s^−1^ seriously underestimates the ER; [[5], [6], [7]], hereafter, we call this method van Gorsel method). This method omits most nighttime data and uses the CO_2_ flux data from observations near sunset, when nighttime advection has not yet affected the EC measurement (Fig. 1b). Nighttime correction is also crucial for the partitioning of NEE into both gross primary productivity (GPP) and ER because the nighttime ER-temperature relationship is extrapolated to estimate the daytime ER [e.g., 8].

The Gwangneung deciduous and coniferous forest sites in Korea (i.e., GDK and GCK, respectively), which are a part of the Korea Flux Monitoring Network (KoFlux), are typical sites located in hilly and complex terrains where these methods are difficult to apply. CO_2_ drainage is generated earlier than the time assumed by the normal methods used (i.e., before sunset), so use of the peak CO_2_ flux under-/overestimated the true ER. Kang et al. [9] developed a site-specific quality control filter to exclude data that has been strongly affected by CO_2_ advection. They calculated the information flow (i.e., transfer entropy, which measures the reduction in uncertainty due to knowledge of another variable, Schreiber [10]) of multi-level CO_2_ concentrations between uphill and downhill sites and identified the timing of CO_2_ drainage based on the significant information flow from uphill (GDK) to downhill (GCK). This site-specific filter, which is qualitatively similar to a hybrid application of the abovementioned two methods, substantially reduced the disagreement among the different traditional methods for nighttime CO_2_ correction. However, this method has low applicability because measurements for the CO_2_ concentration profiles are required from two or more locations, and a series of data recorded over an extended time period is necessary to produce precise results. To overcome these shortcomings, we propose a hybrid of the u* filtering and van Gorsel methods, which can be used with better applicability.

### Measurements and data pre-processing before nighttime CO_2_ flux correction

In both study sites, the EC system was used to measure the eddy CO_2_ fluxes, including u*, from a 40 m tower. The wind speeds and temperatures were measured with a three-dimensional sonic anemometer-thermometer (SAT; Model CSAT3, Campbell Scientific Inc., Logan, Utah, USA), and CO_2_ concentrations were measured with an open-path infrared gas analyzer (IRGA; Model LI-7500, LI-COR Inc., Lincoln, Nebraska, USA). Measurements of EC were made at 30 min intervals, and associated statistics were calculated online from the 10 Hz raw data and stored in dataloggers (Model CR5000, Campbell Scientific Inc.). Other measurements such as net radiation, air temperature, humidity, and precipitation were sampled at intervals of one second, averaged over 30 min, and also logged in the dataloggers (Model CR3000 for the GDK site and CR1000 for the GCK site, Campbell Scientific Inc.). Further information regarding the EC and meteorological measurements can be found in Kwon et al. [12], and Kang et al. [13].

Multi-level profile systems were installed to measure the vertical profiles of the CO_2_ concentrations at both sites, and to estimate the storage flux using a closed-path IRGA (Model: LI-6262, LI-COR Inc.). Measurements were made at heights of 0.1, 1, 4, 8 (base of the crown), 12 (middle of the crown), 18 (the canopy top), 30, and 40 m for the GDK site and 0.1, 1, 4, 12 (base of the crown), 20 (middle of the crown), 23 (the canopy top), 30, and 40 m for the GCK site. More information regarding the multi-level profile system can be found in Hong et al. [2] and Yoo et al. [14].

To improve the quality of the data, the collected data were examined with the quality control procedure based on the KoFlux data processing protocol [[15], [16], [17]]. This procedure includes a sector-wise planar fit rotation (PFR; [[18], [19], [20]]), WPL (Webb-Pearman-Leuning) correction [21], a storage flux calculation [22], a spike detection [22], and gap-filling (marginal distribution sampling method [8]).

### General nighttime CO_2_ flux correction and partitioning methods

The KoFlux data processing protocol includes three different nighttime correction methods: the friction velocity (u*) filtering method (FVF), the light response curve method (LRC), and the modified van Gorsel method (VGF) [7,9,16,17]. Unlike FVF and VGF, the LRC method uses daytime CO_2_ flux data and the y-intercept of the light response curve (as the estimated daytime ER), which can be obtained from the regression of downward shortwave radiation and daytime CO_2_ flux. These three filtering methods each have a means of selecting good quality CO_2_ flux data. The site-specific settings for the individual methods were as follows: (1) the lower u* threshold for the FVF was 0.3 m s^−1^ for both the GDK and GCK sites [9,16], (2) the Michaelis-Menten-type light response curve ($\text{NEE}=R_{\text{LRCd}}-(\alpha Q_{t}A_{\max}/\alpha Q_{t}+A_{\max})$, where *R*_LRCd_ is the estimated mean daytime ER, *α* is the apparent quantum yield, *A*_max_ is the canopy scale photosynthetic capacity, and *Q_t_* is the total incident shortwave radiation above the canopy; van Gorsel et al. [7] was used to estimate *R*_LRCd_ for the LRC, and (3) after calculating the median diurnal variation of the observed CO_2_ flux for a certain period, the peak that occurred approximately at sunset (*R*_max_) was directly used for the modified VGF [9,16]. A 30-day moving window was applied to obtain the daily *R*_LRCd_ and *R*_max_.

The selected ER data from each filtering method were processed as follows. First, the parameters in the temperature response function (TRF, Lloyd-Taylor equation, $\text{ER}=R_{ref}\exp(E_{0}(1/T_{ref}-T_{0})-1/(T_{a}-T_{0})))$, where *R_ref_* is the reference ER, *T_ref_* is the reference temperature (=10 °C), *E*_0_ is the activation energy parameter (°C^−1^), *T_0_* is -46.02 °C and *T_a_* is the air temperature (°C), Lloyd and Taylor [23]) were estimated using the selected observed ER. Second, the bad (or missing) data were replaced with the calculated values using the air temperature and the TRF with the estimated parameters. *R_ref_* was estimated using a 30-day moving window. The moving window was shifted every 5-days to consider the variations in an ER controlled by soil moisture and phenology, which are not considered in the Lloyd-Taylor equation. The *E*_0_ is constant for each site-year, which is estimated using the generic algorithm proposed by Reichstein et al. [8] that derives a short-term temperature sensitivity [see 8, 15 for more details]. For the LRC and the modified VGF, the nighttime CO_2_ fluxes were filtered out if the observed nighttime CO_2_ fluxes were underestimated to a result beyond the 95% confidence interval of the ER model (i.e., Lloyd and Taylor equation). Such filtering and corrections were not applied to the daytime CO_2_ fluxes because during this time the atmosphere is unstable and well mixed [e.g., 6]. Instead, the relationships were extrapolated from nighttime to daytime for estimating daytime ER. GPP was calculated by subtracting NEE from ER.

### Site-specific filter

Kang et al. [9] developed the site-specific quality control filter to exclude data strongly affected by CO_2_ advection using the observed multi-level CO_2_ concentrations at the GDK (uphill) and GCK (downhill) sites. The information flow was calculated (i.e., transfer entropy, which measures the reduction in uncertainty due to the knowledge of another variable, Schreiber [10]) for the multi-level CO_2_ concentrations between the uphill and downhill sites and the timing of CO_2_ drainage was identified based on the significant information flow from uphill to downhill. The filter discards the data: (1) when the CO_2_ drainage is entirely generated from the prevailing mountain winds (i.e., 21:00 to 9:00, 8:00, and 7:30 for the dormant, transition, and growing seasons, respectively), (2) when u* is lower than the threshold (0.3 m s^−1^) while the drainage flow is developing (from 17:00 to 21:00), and (3) until the accumulated CO_2_ completely dissipates from the downhill (GCK) site (i.e., before noon).

### The moving point test (MPT) method and its modification

**Fig. 2:**
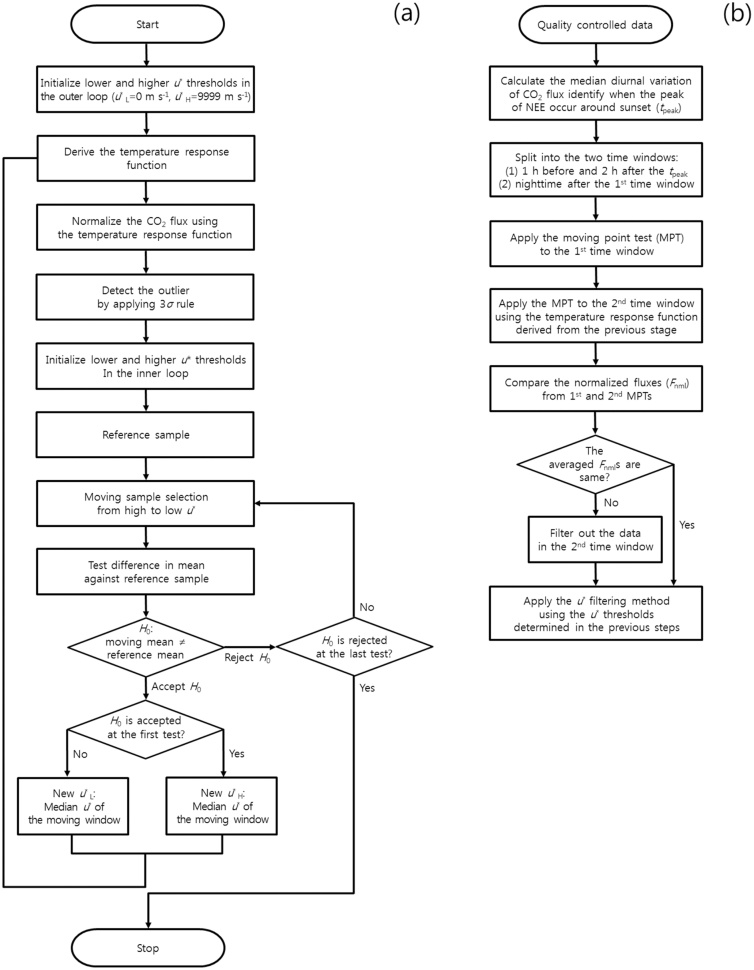
Flowcharts of (a) the original moving point test (MPT) method (adapted from Gu et al. [4]) and the modified MPT method.

The objective of the moving point test (MPT) method is to determine an intermediate range of u* where the night-time CO_2_ fluxes are independent of u* [4]. The method searches for lower and higher u* thresholds, which are found by the statistical testing (i.e., *t*-test) of a group of points with consecutive u* values in a narrow moving window against a reference sample as follows: (1) Initialize the lower and upper u* thresholds (u*_L_ and u*_H_) for the outer loop by setting u*_L_ = 0 and u*_H_ = 9999. (2) Derive the TRF for ER (i.e., Lloyd-Taylor equation) through regression using data with u* values between u*_L_ and u*_H_. The outer loop starts from this step. (3) Normalize the CO_2_ flux using the TRF (i.e. divide the measured flux by the value estimated from the TRF). (4) Detect and remove the outliers by applying the 3*σ* rule (*σ* is the standard deviation), and sort the data in ascending order of u*. (5) Initialize the u*_L_ and u*_H_ for the inner loop by setting u*_L_ = 0 and u*_H_ = 9999. (6) Filter the data using u*_L_ and u*_H_ as the reference sample. The inner loop starts from this step. (7) Start from the point with the highest u* among the remaining data and take *n* points with consecutive u* values. This group of points is called the moving sample. (8) Compare the mean normalized flux of the moving sample (*F*_m_) to the mean normalized flux of the reference sample (*F*_r_) using a statistical *t*-test with the null hypothesis *H*_0_: *F*_m_ ≠ *F*_r_. (9) If *H*_0_ is rejected, return to Step 7 and iterate the steps using the data point adjacent to the previous starting point (i.e., the next highest u* group). (10) If *H*_0_ cannot be rejected, update the median u* of the moving sample as the new u*_L_. (11) If *H*_0_ cannot be rejected at the first test, update the median u* of the moving sample as the new u*_H_. (12) If u*_L_ or u*_H_ are updated, return to Step 2 for the next round of outer loop iteration. (13) If *H*_0_ is rejected in the last test, the two thresholds are determined (i.e., the last updated u*_L_ and u*_H_). For applying the MPT method, there were two criteria, i.e., the significance level in the *t*-test (*α*_MPT_) and the size (i.e., the number of data) of the moving sample (*n*). According to Gu et al. [4], the *α*_MPT_ is 0.1, and the *n* is 25. The MPT method was applied for periods of three months. Details regarding the MPT method are described in the flowcharts (Fig. 2 a) and from Gu et al. [4].

The original MPT method excludes night data when the median u* were lower than the lower u* threshold at Steps 2 and 7, to avoid an underestimation of the CO_2_ flux due to drainage flow. However, this consideration could be inappropriate for sites with hilly terrain that are usually affected by drainage flow (e.g., the study sites, GDK and GCK), resulting in CO_2_ fluxes that are either close to 0 mg CO_2_ m^−2^ s^−1^ and/or much smaller than the true values during most of the night, except near sunset. Occasionally at night, the mean diurnal variation in CO_2_ flux during the growing season, under high u* conditions (>0.3 m s^−1^) for the sites, has values of ˜ 0 mg CO_2_ m^−2^ s^−1^ [see Fig. 5 in 9].

Thus, we modified the original MPT method to apply it to hilly terrain sites by: (1) splitting it into two time windows, i.e., the time window near sunset (when drainage has not yet fully manifested) and the subsequent time window immediately after the first, (2) applying the MPT method to each time window, (3) comparing the results between the two time windows and determining the significance of CO_2_ drainage at midnight by checking whether the mean normalized nighttime CO_2_ fluxes for the two time windows were significantly different, and (4) excluding all the data in the second time window if the CO_2_ drainage is significant, and then applying the FVF method for both time windows using the u* thresholds determined in the previous steps.

The best feature of the modified MPT method is that the time is split into the two time windows based on van Gorsel et al. [7]: (1) calculating the median diurnal variation of the CO_2_ flux and identifying when the peak of NEE occurred approximately at sunset; (2) splitting the time windows, i.e., the first window one and two hours before and after the time of peak occurrence, and the second window in the time immediately after the first time window, respectively. This method assumes that there are no significant differences in the biological and meteorological conditions affecting the ER between the two time windows. At the study sites, the mountain wind rose continuously at night, and we could not find any parameter that would make a difference to the ER between the two time windows, except for ecosystem temperature on a short time scale. Thus, we compared the averages after normalizing the flux measurements using the same TRF as that used in the original MPT method proposed by Gu et al. [4].

### Test of the modified MPT

**Fig. 3:**
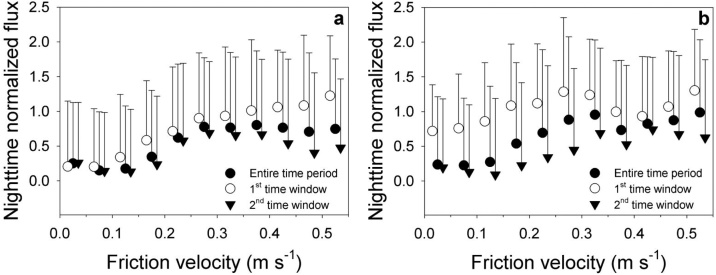
The dependencies of nighttime CO_2_ flux on friction velocity (u*) at the (a) GDK and (b) GCK sites during the growing season (DOY 121–300 for the GDK and DOY 91–300 for the GCK) for the entire time period, the first time window (near sunset), and the second time window (i.e., after the first time window). The nighttime CO_2_ fluxes are normalized by the estimated ecosystem respiration using the temperature response function (i.e., Lloyd and Taylor equation, Lloyd and Taylor [23]). The parameters of the temperature response function are estimated using the observed flux data when the value for u* is higher than the threshold of 0.3 m s^−1^. The error bars indicate the standard deviation of the normalized flux for each u* bin.

Before testing the modified MPT method, we checked the dependency of the nighttime CO_2_ flux on u* for each time window (Fig. 3). If the FVF and VGF methods work normally for the sites, then the mean normalized fluxes are almost the same regardless of the time windows when u* is higher than a threshold and the mean normalized fluxes rarely depend on u* in the case of the first time window, respectively. However, sometimes both conditions were not sufficiently satisfied for the sites because the mean normalized fluxes were significantly different between the two windows in some u* bins (i.e., underestimation for the second time window) and the fluxes still depended on u* for the first time window. The former indicates that the value for u* measured at 40 m could not reflect the turbulent conditions below the canopy due to the decoupling of the atmosphere above and below the canopy (i.e., drainage). Overall, these results emphasize the necessity for a modification to the traditional nighttime correction methods for these types of site.

**Fig. 4:**
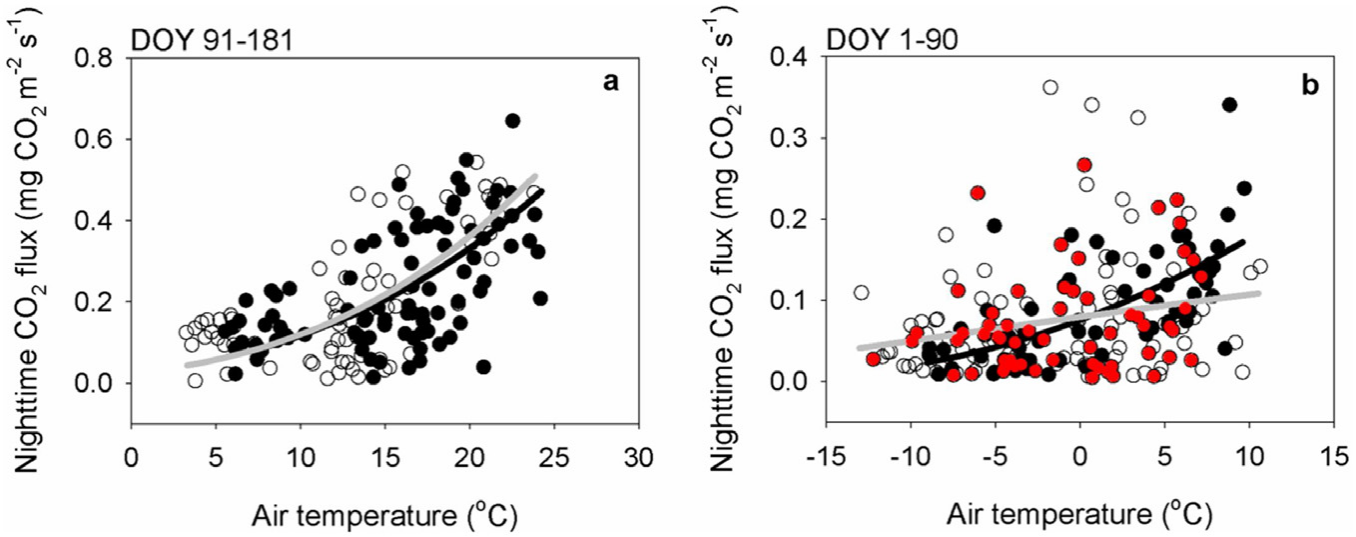
The relationship between the nighttime CO_2_ flux and air temperature after applying the modified moving point test (MPT) (filtered by the u* thresholds which are determined from the modified MPT) for the GCK in 2008, DOY 91–181 (a) and DOY 1–90 (b). The black color indicates the first window of time, whereas the white color indicates the second time window. The red color indicates that u* was higher than 0.3 m s^−1^ (i.e., the u* threshold which is determined by the traditional method) among the second time window data. The solid black line indicates the fitting line of the Lloyd-Taylor equation [23] using the filtered data of the first time window, whereas the solid gray line indicates that of the second time window.

Fig. 4 shows some of the results from using the MPT method at the GCK site in 2008. During DOY 91–181, the mean normalized nighttime CO_2_ fluxes after u* filtering for both time windows were not statistically different (Fig. 4a). This result suggests that there was no drainage, and/or the drainage effect was negligible at midnight. Meanwhile, during DOY 1–90, there are obvious significant differences, suggesting that the observation for the second time window cannot represent the true ER due to drainage (Fig. 4 b). A significant number of results from the second time window are near to 0 mg CO_2_ m^−2^ s^−1^ although the air temperature was higher than 0 °C (and the u* is higher than the traditional u* threshold, i.e., 0.3 m s^−1^). Consequently, the fitting lines of the Lloyd-Taylor equation using the filtered data are significantly different. This implies that (1) the observed CO_2_ fluxes can underestimate the true ER for the second time window despite a high value of u*, and (2) such underestimation can hinder a reliable determination of the u* threshold (and therefore the optimization of the parameters for the ER equation).

**Fig. 5:**
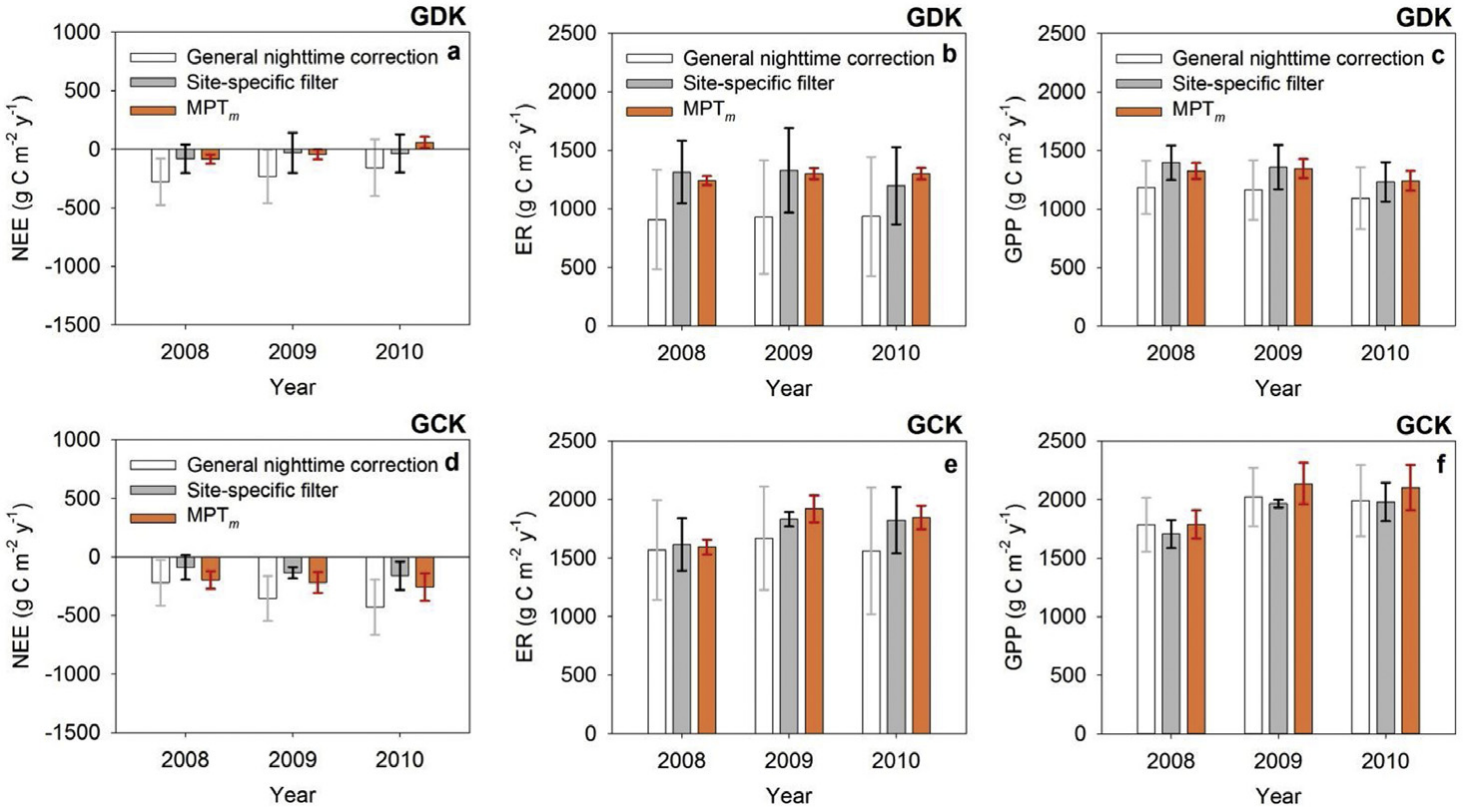
The (averaged) annual CO_2_ budgets (NEE: net ecosystem exchange, GPP: gross primary production, ER: ecosystem respiration) from the three traditional nighttime correction methods (i.e., the u* filtering method, light response curve method, and van Gorsel method) (a) without and (b) with the application of the site-specific filter (adapted from Kang et al. [9]) and from the modified MPT (MPT*_m_*) method for both sites. The gray and black error bars indicate the standard deviation of the results from the three general nighttime correction methods, whereas the red error bars indicate the random uncertainties in the annual CO_2_ budgets from MPT*_m_* (quantified according to Finkelstein and Sims [24], and Richardson and Hollinger [25]).

The results from the modified MPT method are summarized in Table 1. It is hard to find a particular characteristic that leads to the drainage effect, implying that the drainage effect is a consequence of the complex influence of micrometeorology, phenology, data availability, etc. Such results require careful examination with micrometeorological and ecological perspectives using other independent observations because the modified MPT method is an empirical method, as are the other traditional correction methods.

**Table 1:** Summary results generated from the modified moving point test (MPT) for the GDK and GCK sites. u*_L_ and u*_H_ indicate lower and higher u* thresholds, 1^st^ and 2^nd^ indicate the first and second time windows, respectively. The significance of CO_2_ drainage at midnight was determined by checking that the mean normalized nighttime CO_2_ fluxes for the two time windows were significantly different.

Site	Year	DOY	u*_L_ - 1^st^	u*_H_ - 1^st^	u*_L_ - 2^nd^	u*_H_ - 2^nd^	Drainage effect at midnight
GDK	2008	1-90	0.179	9999	0.337	9999	negligible
		91-181	0.215	9999	0.363	9999	significant
		182-273	0.178	9999	0.215	9999	negligible
		274-366	0.144	0.271	0.171	9999	negligible
	2009	1-90	0.216	9999	0.411	9999	negligible
		91-181	0.406	9999	0.169	9999	significant
		182-273	0.238	9999	0.156	9999	significant
		274-365	0.338	9999	0.184	0.506	significant
	2010	1-90	0	9999	0.326	0.47	negligible
		91-181	0	9999	0.202	0.341	significant
		182-273	0.247	9999	0.173	9999	negligible
		274-365	0.166	9999	0.255	9999	negligible
GCK	2008	1-90	0.409	9999	0.174	9999	significant
		91-181	0.29	9999	0.25	9999	negligible
		182-273	0.164	9999	0.221	9999	negligible
		274-366	0.149	9999	0.197	9999	negligible
	2009	1-90	0.248	9999	0.425	9999	negligible
		91-181	0.252	9999	0.122	9999	significant
		182-273	0.171	9999	0.141	0.198	significant
		274-365	0.232	9999	0.238	9999	negligible
	2010	1-90	0.157	9999	0.295	9999	significant
		91-181	0.157	9999	0.137	9999	significant
		182-273	0.125	9999	0.122	0.216	significant
		274-365	0.098	0.417	0.348	9999	significant

It is quite challenging to validate the method without other independent measurements using different approaches such as the chamber method [e.g., [[5], [6], [7]]]. There are few sites where chamber systems are used to measure CO_2_ fluxes for an entire ecosystem. Thus, we checked the validity of the proposed method by comparing our results with those from previous study using the site-specific filter for drainage [9, see section ‘Site-specific filter’]. Fig. 5 shows the (averaged) annual CO_2_ budgets from the three traditional nighttime correction methods (see the section ‘General nighttime CO_2_ flux correction and partitioning methods’) with and without applying the site-specific filter, and those from use of the modified MPT method. We already knew that the site-specific filter improved on the discrepancies (see the standard deviation among the results) between the three nighttime correction methods and the underestimation of ER [9]. On comparing the results from the modified MPT method with those obtained after applying the site-specific filter, it was found that almost all the results agree with each other within the margin of error. This partially validates the modified MPT method. It is noted that the values for NEE and GPP at the GCK site from the modified MPT method were slightly overestimated due to the daytime (not nighttime) filtering (i.e., the third criterion of the site-specific filter).

The distinctive feature of the new technique proposed in this study for nighttime flux filtering (and correction) of eddy CO_2_ fluxes is that two existing methods were merged to produce a new method. The u* filtering (i.e., original MPT method) and van Gorsel methods have been merged to produce the modified MPT method. Such a strategy enhances the strengths and makes up for the weakness of the original methods. There are other examples of such improvements; the lookup table method and mean diurnal variation method were merged into the marginal distribution sampling method for flux gap-filling [25], and the marginal distribution sampling method and the simplified Rutter spars model were merged into the model–statistics hybrid method for gap-filling and partitioning of water vapor fluxes [17]) The modified MPT method can (1) filter out the underestimated CO_2_ fluxes near sunset using u* and (2) salvage the omitted data from most of the nighttime when using the van Gorsel method. Notably, the modified MPT method for nighttime CO_2_ flux correction substantially improves its applicability, leading to the expectation that it will contribute to the standardization of eddy covariance data processing.

## Appendix A Supplementary data

MATLAB script for applying the method and sample data.
